# Aluminothermic Reduction of Manganese Oxide from Selected MnO-Containing Slags

**DOI:** 10.3390/ma14020356

**Published:** 2021-01-13

**Authors:** Artur Kudyba, Shahid Akhtar, Inge Johansen, Jafar Safarian

**Affiliations:** 1 Department of Materials Science and Engineering, Norwegian University of Science and Technology (NTNU), Alfred Getz Vei 2, 7034 Trondheim, Norway; jafar.safarian@ntnu.no; 2 Hydro Aluminum, Romsdalsvegen 1, 6600 Sunndalsøra, Norway; shahid.akhtar@hydro.com (S.A.); inge.johansen@hydro.com (I.J.)

**Keywords:** aluminothermic reduction, Al dross, FeMn, slag

## Abstract

The aluminothermic reduction process of manganese oxide from different slags by aluminum was investigated using pure Al and two types of industrial Al dross. Two types of MnO-containing slags were used: a synthetic highly pure CaO-MnO slag and an industrial high carbon ferromanganese slag. Mixtures of Al and slag with more Al than the stoichiometry were heated and interacted in an induction furnace up to 1873 K, yielding molten metal and slag products. The characterization of the produced metal and slag phases indicated that the complete reduction of MnO occurs via the aluminothermic process. Moreover, as the Al content in the charge was high, it also completely reduced SiO_2_ in the industrial ferromanganese slag. A small mass transport of Ca and Mg into the metal phase was also observed, which was shown to be affected by the slag chemistry. The obtained results indicated that the valorization of both Al dross and FeMn slag in a single process for the production of Mn, Mn-Al, and Mn-Al-Si alloys is possible. Moreover, the energy balance for the process indicated that the energy consumption of the process to produce Mn-Al alloys via the proposed process is insignificant due to the highly exothermic reactions at high temperatures.

## 1. Introduction

Aluminum is the most abundant metallic elements in the Earth’s crust, posing an excellent combination of chemical, mechanical, and physical properties which makes it suitable for many applications [1]. Aluminum is mainly produced by two different methods: (I) a primary aluminum production from bauxite ore by the Bayer process for alumina extraction followed by Hall–Heroult electrolysis for Al extraction from alumina, and (II) by recycling aluminum from process scrap and wasted aluminum products [2,3,4]. In 2019, the global production of metallic aluminum was approx. 64 million metric tons, with a daily average of 174.5 thousand tons [5]. As a result of the exposure of liquid aluminum to the oxidizing atmosphere that is present during the process of melting and alloying, a surface oxidation takes place, leading to the formation of a semisolid skin over the molten Al metal, which also hinders further oxidation. This floating skin over liquid Al is called aluminum dross and consists mainly of aluminum oxide, metallic aluminum, magnesium spinel, periclase, and quartz [6,7]. There are two types of Al dross: (I) White Dross (the primary dross), which is formed during the primary production of aluminum (mainly aluminum ingots), containing approximately 15–80% metallic aluminum, 20–85% aluminum oxide, and 5% salts, and (II) Black Dross (the secondary dross), which is a by-product of the secondary production of aluminum, containing 7–50% metallic aluminum, 30–50% aluminum oxide, and 30–50% salt flux [8,9,10,11,12]. Each year, the world aluminum industry produces approximately four million tonnes (Mt) of Aluminum White Dross (AWD) and more than a million tonnes of Aluminum Black Dross (ABD), and around 95% of this material (ABD) is landfilled [4,13]. Aluminum dross is a potential toxic industrial waste inevitably generated in aluminum smelter plants. The safe disposal of Al dross as a waste is a burden to the aluminum industry because of the effects of improper disposal on the eco-system. Owing to the large annual production of Al dross and its environmental and economic impacts, aluminum dross undergoes industrial treatments to extract valuable products, including metallic aluminum. Two methods of Al dross treatment are used: (I) a pyrometallurgical route, which is a conventional method of treating Al dross, liberating metallic aluminum in the liquid state, and (II) a hydrometallurgical route, which involves the extraction of metallic aluminum from the Al dross by converting it into aluminum salts and compounds. The recycling of aluminum dross is crucial for environmental protection, economic reasons, and sustainable development with regard to circular (“zero waste”) economy.

Manganese is ranked as the 12th most abundant element in the Earth’s crust, is applied in the steel and aluminum industry, and in its elemental and alloy forms is used as an alloying element. The massive production of manganese is via the carbothermic reduction of Mn ores in submerged arc furnaces, which yields Mn ferroalloys such as high-carbon ferromanganese (HCFeMn) and silicomanganese, SiMn [14]. In 2019, about 4.4 and 7.7 Mt of HCFeMn and SiMn were produced, respectively. In addition, 1.43 and 1.62 Mt of refined ferromanganese and manganese metal were fabricated [15]. A by-product of the HCFeMn process is a slag that contains a considerable amount of MnO, usually in the range of 20–45% MnO, along with other oxides such as CaO, MnO, MgO, Al_2_O_3_, and SiO_2_ [16]. If HCFeMn slag is not utilized in SiMn production, it is landfilled or used in other industries. It contains a significant amount of Mn element and its valorization to extract Mn is also important from the circular economy point of view.

A literature survey [17,18,19,20,21,22,23,24] revealed a few works on the aluminothermic reduction of MnO by dissolved Al in a continuous galvanizing bath. However, in the available literature there is a limited number of studies analyzing the results of the aluminothermic reduction of manganese oxide using real industrial materials such as Al dross or FeMn slag. Dávila et al. [17] investigated the effect of magnesium concentration in the molten aluminum produced from beverage cans on the process of the aluminothermic reduction of Mn_2_O_3_ particles obtained from the cathodes of discharged alkaline batteries. The authors have proven that the magnesium content of the base alloy is a very important factor in the aluminothermic reduction, since this element improves the wettability of aluminum on the Mn_2_O_3_ particles, which in turn supports solid/liquid reaction. Kavitha and McDermid [18] have investigated the aluminothermic reduction of MnO by dissolved Al in the continuous galvanizing process. They conclude that the MnO reduction reaction is a relatively simple dissolution reaction in which the composition of the MnO layer was not altered during the reaction. Furthermore, the thickness of the thin Al_2_O_3_ reaction product was relatively constant (3–4 nm) for all the reaction times investigated. Jiaxing et al. [22] studied the aluminothermic reduction of pure MnO_2_ by metallic Al particles through a thermite process, and studied the conversion of MnO_2_ to Mn_2_O_3_ and Mn_3_O_4_ using Differential Scanning Calorimetry (DSC) techniques. They have provided information about the heat generation due to the reactions at temperatures below 900 °C, but they have not produced metallic manganese in their experiments. Sarangi et al. [23] studied the reaction between MnO_2_ and Al powders using the Differential Thermal Analysis (DTA) technique and determined the heat generation due to aluminothermic reduction reactions, varying the MnO_2_/Al ratio. Based on the rate of heat generation in DTA experiments, they calculated the rate of the reduction reaction and further performed a kinetic study. Bhoi et al. [24] studied the aluminothermic reduction of a manganese ore particles by Al powder to produce ferromanganese, and used lime and fluorspar in their mixtures. They performed reduction reactions via roasting at moderate temperatures of 650 °C and 950 °C, and produced ferromanganese samples with 70–80 wt.% Mn and 12–16 wt.% Fe. In all these studies, pure Al powders were used, and pure MnO_2_ or manganese ore were applied as the manganese oxide source [22,23,24]. The novelty of the present study is established by following features: (1) the Al reactant is not a pure powder, but rather industrial Al dross; (2) the source of manganese oxide is not pure manganese oxide powder, but rather industrial ferromanganese slag, which is a complex material; (3) the previous researchers applied the thermite process, in which the mixtures of powdered materials are heated to moderate temperatures, usually below 1000 °C, while in the present study we heat up the reactants to much higher temperatures to obtain all the charged mixtures in a molten state; (4) the present work is more oriented towards an evaluation of the products, and the target product is Al-Mn alloy, not Mn oxide or ferromanganese.

As is mentioned above, the present work is focused on the high-temperature aluminothermic reduction process of MnO from synthetic and industrial HCFeMn slags by pure Al and two industrial AWDs. The main chemical reaction of the process at elevated temperatures can be written as:

3MnO + 2Al = 3Mn + Al_2_O_3_   ΔH (1500 °C) = −466.5 kJ/mol. (1)

Obviously, the highly exothermic nature of this reaction is beneficial regarding the overall process energy consumption. Moreover, thermodynamic software (FactSage ver. 7.3.) is used to discuss the results and find the mass and energy balances.

## 2. Experimental Procedure

The materials preparation and applied methodology are described as follows.

### 2.1. Material Preparation

Two aluminum dross samples were collected from skimmed dross over the surface of molten primary Al and an Al-Si-Mn alloy (grade series 1000 and 4000, respectively). In order to directly extract a representative Al dross sample from the surface of the molten alloy, a special sampling tool was designed and developed. Initially, the sampling tool was introduced into a dross tub, and when the Al dross was skimmed from the reverberatory furnace into the tub in which the tool was positioned in, a portion of the hot dross was collected from the surface of the molten alloy by the sampling unit. The thickness of the dross layer collected for testing was in the range of 20–30 cm. The dross 1 (from the primary Al) was used in its original form, and the dross 2 (from the Al-Si-Mn alloy) was subjected to a mechanical ball milling at room temperature to separate the fine oxide and the inclusion of the dross and to obtain rich metallic Al-containing particles. After the mechanical treatment, the milled dross 2 was partitioned by sieving and a particle size of 1.25–2 mm was further used. The microstructures and characteristics of these dross samples were studied by the Zeiss Ultra 55 Scanning Electron Microscope (SEM, Carl Zeiss Microscopy GmbH, Jena, Germany), coupled with energy-dispersive X-ray spectroscopy (EDS) and X-Ray Diffraction (XRD) (Bruker D8 A25 DaVinci X-ray Diffractometer with CuKα radiation with LynxEye™ SuperSpeed Detector [25,26] (Bruker Corporation, Billerica, MA, USA). In this study, a high-purity Al metal (99.9%) was used for a trial.

**Figure 1:**
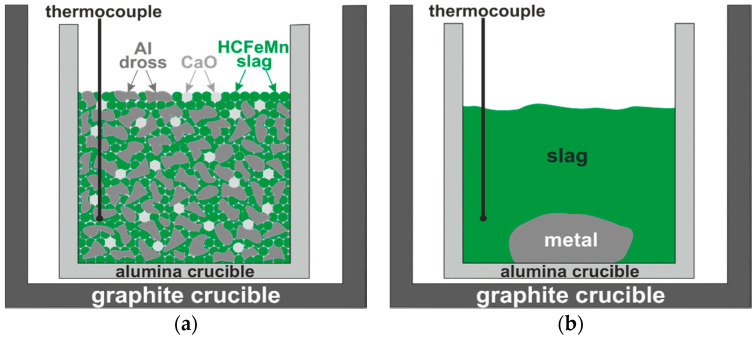
A scheme of (**a**) charged materials for exp. 3 in crucible before the reaction test, and (**b**) the produced slag and metal after reactions.

Two types of MnO-containing slags were used in this study: a binary CaO-MnO synthetic slag and an industrial HCFeMn slag received from the industry, which was characterized by PANalytical Zetium 4 kW X-ray Fluorescence (XRF, Malvern Panalytical Ltd, Malvern WR14 1XZ, UK). The synthetic slag was made by mixing pure CaO and MnO (above 99% purity level) oxides, and then melting in a top open induction furnace at 1873 K in a graphite crucible to obtain a CaO 25 wt.%. Pure Al and the dross 1 particles (1–10 mm) were mixed with the synthetic slag. The dross 2 (1.25–2 mm) was mixed with the HCFeMn slag, whereas CaO was in addition added with masses of about 10% and 25%. The addition of Al reductants to reduce the synthetic slag was required two times for the reduction of all MnO in the slag, and the utilized mass of dross 1 was approximated 69 g, as it contains a significant amount of Al_2_O_3_, which was estimated to be 34 wt.%, while for the HCFeMn slag the same masses amount of treated dross 2 was used. The charge mixture details are given in Table 1. The mixtures were charged into alumina crucibles, and then they were put in graphite crucibles, as is schematically shown in Figure 1a.

### 2.2. Aluminothermic Reduction

The crucibles with the charge mixtures were put in an induction furnace and a thermocouple was put into the charge mixture to measure the temperature inside the crucible. Each crucible was heated at about a 293 K/min rate up to 1773 K and held for 30 min in a protective atmosphere (flow gas Ar 5.0 at a constant pressure of 1030 mbar). The temperature recordings indicated the reaction kinetics, and it was observed that, upon reaching the target temperature (1773 K) for a few minutes, the temperature in the crucible was rapidly increased to a maximum in the range of 1973–2073 K, indicating that an aluminothermic reaction (1) was rapidly taking place. After that, the temperature inside the crucible declined to the target temperature. Then, the furnace power was turned off and consequently the molten components solidified. The metal is much heavier than the slag and it sinks in the crucible, as schematically illustrated in Figure 1b.

The produced metal and slag were separated after breaking the crucibles, and metallography samples were produced from them via mounting in epoxy resin followed by grinding and metallographic polishing. The structure and chemical composition were characterized using a Zeiss Ultra 55 SEM coupled with EDS.

## 3. Results and Discussion

The obtained results for the materials and products are presented and discussed as follows.

### 3.1. Reactant Materials Characteristics

**Figure 2:**
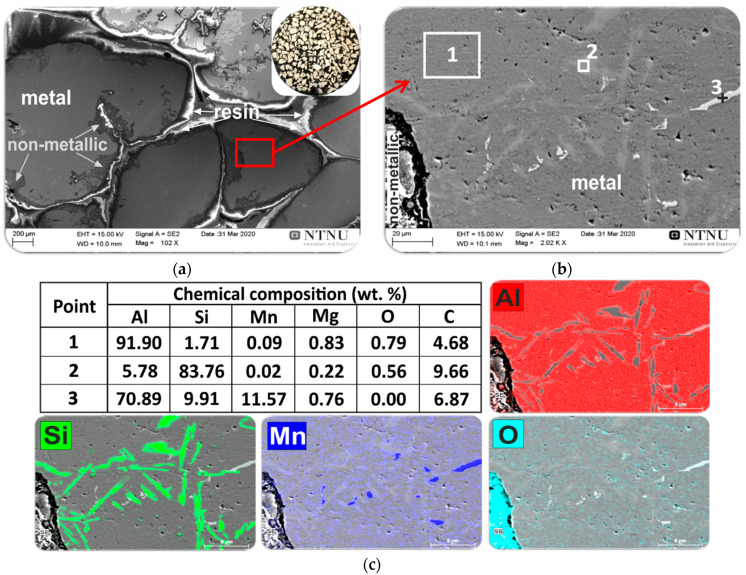
The SEM/EDS microstructural analysis results of particles of Al dross of size 1.25–2 mm used in the aluminothermic reduction: Scanning Electron Microscope–Backscattered Electrons images (**a**,**b**), EDS mapping (**c**).

**Figure 3:**
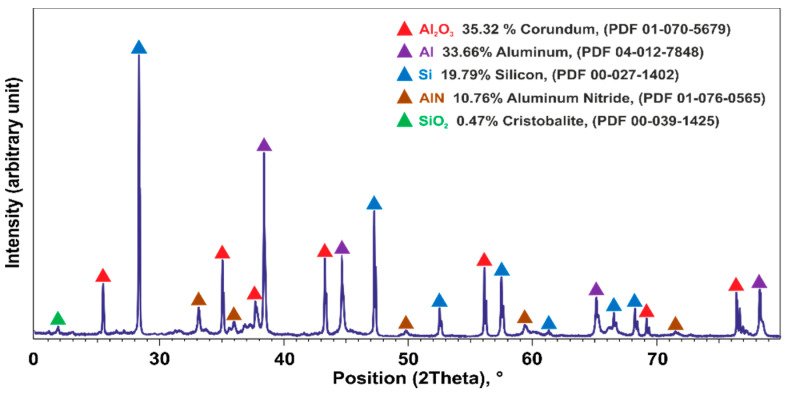
XRD pattern of the fine dross (<1 mm) that contains non-metallic components.

The results of the SEM and EDS microstructural analyses of the separated Al dross particles with 1.25–2 mm sizes that were later used in the aluminothermic reduction are shown in Figure 2. Figure 2a shows a few particles (mounted in resin) and all the particles show two main components: a major metallic portion and a darker non-metallic portion. A microstructural analysis of the particles with a size below 1.25 mm indicated a significantly higher non-metallic portion as compared to that of the larger particles. The elemental X-ray mapping of the main elements in Figure 2 shows clearly these dross parts. A semiquantitative XRD analysis of the fine particles under 1 mm in Figure 3 indicated that about 50% of the material is Al_2_O_3_, AlN, and SiO_2_, and the rest is metallic Al and Si. Hence, in the utilized 1.25–2 mm particles, we may have the same compounds but in a smaller total amount of about 18%, as approximated by image analysis. Hence, the metallic portion of these particles is about 80%. The SEM microstructural study of dross 1 indicated that almost 2/3 of the material was metallic and the rest was aluminum oxide, and this was the basis for using more mass of dross 1 in experiment 2 compared to experiment 1 (Table 1).

It was found that the metallic portion of the Al dross particles has three main phases: an Al matrix that contains large and small Si particles (area 1 in Figure 2b), an area that contains fine and large Si particles (area 2 in Figure 2b), and an Mn-rich phase (point 3 in Figure 2b). In area 2 in Figure 2b, the Si phase was identified to contain around 84 wt.% Si; however, as Al has insignificant solubility in Si, we may conclude that the detected Al is from the matrix and this phase is pure Si.

The results of the XRF chemical analysis of HCFeMn slag are presented in Table 2. It was found that the dominant component is manganese oxide (46 wt.%). The second phase identified in the material is SiO_2_, which constitutes about 19 wt.%, followed by CaO and Al_2_O_3_, which are around 14 and 11 wt.%, respectively. The slag contains minor amounts of K_2_O, BaO, SO_3_, Na_2_O, TiO_2_, and SrO. As the material is smelted at elevated temperatures, the form of the slag phases in solid state is not important and XRD analysis was not necessary in this study.

### 3.2. Characteristics of the Products

The obtained results were studied regarding the characteristics of the reactants in the experiments in the following.

#### 3.2.1. Interaction of Pure Al with Synthetic Slag

**Figure 4:**
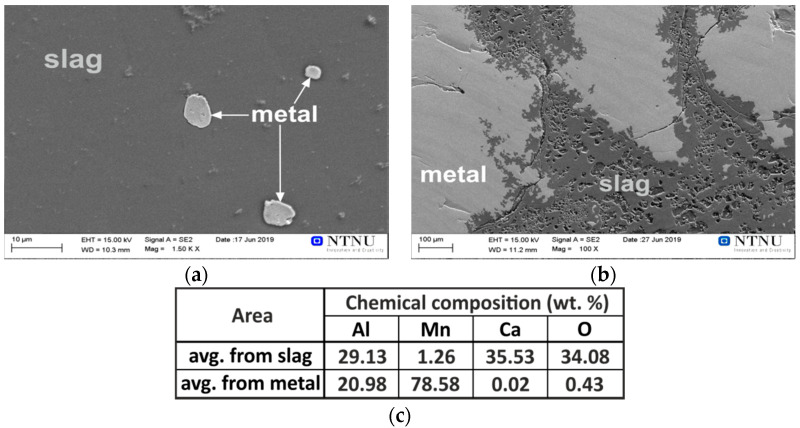
The SEM/EDS microstructural analysis of the cross-section of exp. 1 sample from slag area (**a**) and metal-slag contact area (**b**); EDS (**c**).

The metal and slag phases produced via the pure Al interaction with the synthetic slag were easily separated after breaking the crucible. They were characterized by SEM (with EDS analysis), and it was found that small amounts of metal exist in the slag in the form of tiny solidified metal droplets, as illustrated in Figure 4a. This may be due to the short processing time, so that the complete separation of the heavier metal droplets and their settling due to them having a higher density than the slag has not occurred. As these tiny metal particles are insignificant in mass (less than 1%) compared to the main separated metal phase in the bottom of the crucible, we can conclude here that the metallic product is easily separable from the slag. A typical SEM image from the metal/slag interfacial area is given in Figure 4b, and selected areas were analyzed by EDS. The chemical composition was measured for five different large areas and averages were determined. Obviously, the brighter light grey phase is metallic, with an average of about 79 wt.% Mn and 21 wt.% Al.

The small amount of oxygen can be due to the surface oxidation of the metal phase during the sample preparation, and smaller amount may be in the form of dissolved oxygen in the alloy. Regarding the binary Mn-Al phase diagram [27] and the concentration of the produced metallic alloy, the metal consisted of a solid solution of Al in Mn, βMn, with the highest solubility of Al in it. The dark grey phase in the SEM image of Figure 4 shows that it is a slag phase that consists of Ca, Al, and O elements. The results of local chemical composition analyses by EDS from three different slag areas revealed that it is composed of an average of 35.5 wt.% Ca, 34 wt.% O, and 29.1 wt.% Al. According to the CaO-Al_2_O_3_ phase diagram, the slag contains a fine structure of 3Ca·Al_2_O_3_ and 12CaO·12Al_2_O_3_ phases.

**Figure 5:**
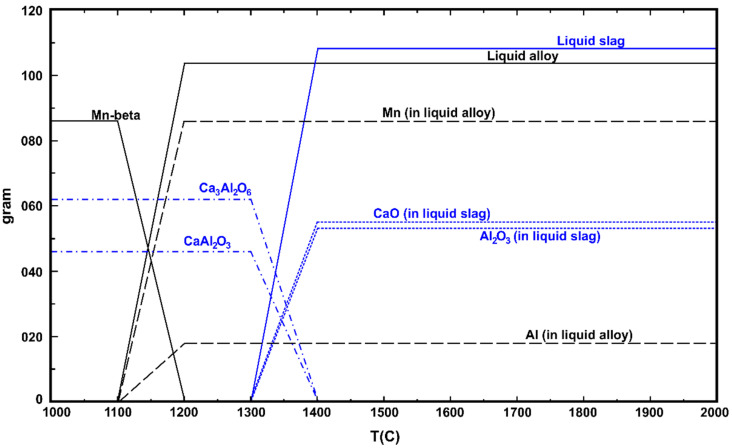
Calculated equilibrium conditions for the interaction of 46 g Al with a 55 g CaO-111 g MnO slag (FactSage ver. 7.3.).

The equilibrium in the interacting system at the process temperature was studied by the FactSage thermodynamic software, version 7.3., using the FACT Oxid and FACT Lite databases [28,29,30,31]. Figure 5 shows that if we interact a binary CaO-MnO mixture with Al, calcium-aluminate slags are produced through the formation of Al_2_O_3_ through the reaction in Equation (1), and its further reaction with the adjacent CaO. Accordingly, metallic Mn is produced, and if there is excess aluminum for Mn oxide reduction it yields a Mn-Al alloy.

The microstructural characterization evaluation is in good agreement with the equilibrium calculations by FactSage (Figure 5) that show the formation of calcium-aluminate slag with an Mn-Al alloy. The same experimental result as the theoretical equilibrium calculations indicates that the applied aluminothermic process is quite fast and has reached almost equilibrium within very short reaction times.

#### 3.2.2. Interaction of Al Dross with Synthetic Slag

**Figure 6:**
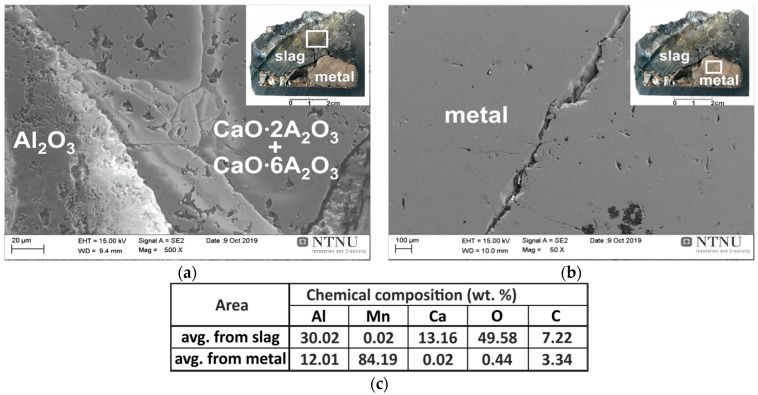
The SEM/EDS microstructural analysis of the cross-section of exp. 2 sample from slag area (**a**) and metal area (**b**); EDS (**c**).

Figure 6b shows the SEM examination results for the produced metal phase through the interaction of the synthetic slag and Al dross 1 in exp. 2. The results of the metal chemical composition analyses show that it consisted of βMn (solid solution of Al in Mn) with an average of about 84 wt.% Mn and 12 wt.% Al. The chemical composition was measured over five different large areas, and averages were then calculated.

The obtained metal in exp. 2 is richer in Mn compared to exp. 1, and this is due to the lower amount of metallic reactant in the utilized dross 1 than in our estimated metallic Al above, yielding less Al in the produced Mn metal. It is worth mentioning that investigating the slag phase indicated that there was no unreacted metallic Al left, showing the formation of completely molten phases at elevated temperatures, complete chemical reactions (1), and proper phase separation. The SEM study of the slag in exp. 2 indicated again a calcium-aluminate slag that contained significant amounts of CaO·2Al_2_O_3_ and CaO·6Al_2_O_3_ phases and a small amount of Al_2_O_3_ phases, which is expected regarding the binary CaO-Al_2_O_3_ slag system, and it having a higher amount of Al_2_O_3_ in the system compared with exp. 1. Similar to exp. 1, in exp. 2 Mn-free calcium aluminate slag was produced again, indicating the complete conversion of MnO to metallic Mn. It is worth mentioning that we did not observe the dissolution/degradation of the alumina crucible, as the molten slag is enriched rapidly by Al_2_O_3_ and the retention time is short.

#### 3.2.3. Interaction of Al-Dross with FeMn Slag

**Figure 7:**
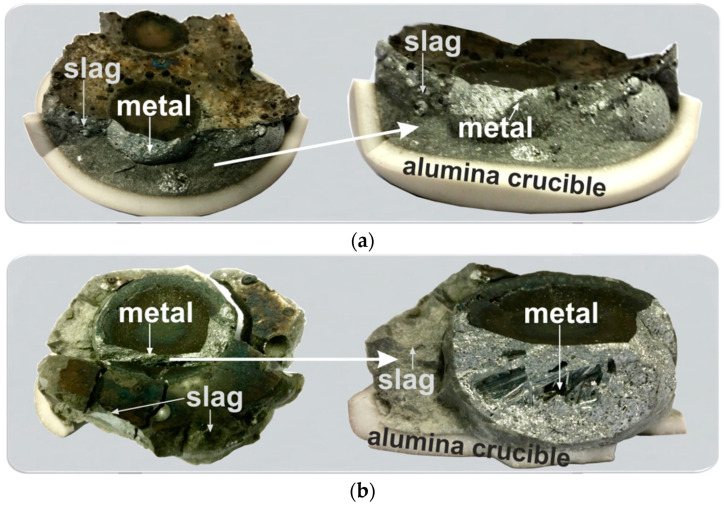
The broken crucibles after experiments 3 (**a**) and 4 (**b**).

The results for the interaction of Al dross with HCFeMn slag indicated that in both exps. 3 and 4 we obtain two separable metal and slag phases, as seen in Figure 7. However, it was found that for the exp. 4 with a higher amount of added CaO, we obtain a single large metal, while for the exp. 3 we have some metal balls in the slag with different sizes. This may indicate that the viscosity of the produced slag in exp. 4 is lower than that of exp. 3, and so the metal droplets join more easily.

**Figure 8:**
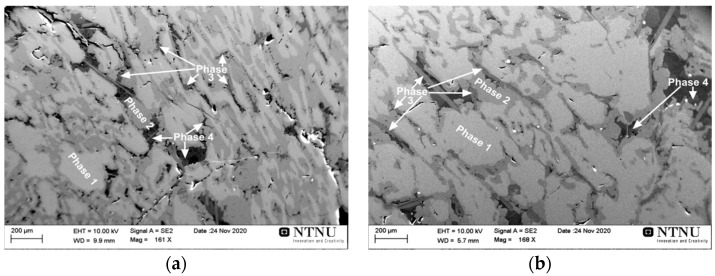
The SEM/EDS microstructural analysis of the cross-section of the metal produced in exp. 3 (**a**) and exp. 4 (**b**).

The microstructural analyses of the produced metals in exp. 3 and exp. 4 show that they have very close chemical compositions and that both metal products are ternary Al-Mn-Si alloys with small amounts of Fe, Mg, and Ca, as illustrated in Figure 8 and Table 3. The existence of Fe in the alloys is obviously due to the aluminothermic reduction of iron oxide from the FeMn slag, as its reduction is possible via chemical reaction (2) as we do not have Fe in the Al dross. Moreover, the chemical analysis of the produced slags indicated that there are calcium aluminate slags (about 99%) with minor amounts of SiO_2_ and MgO. This indicates that almost all the SiO_2_ in the FeMn slag (about 20% in Table 1) has been reduced via the chemical reaction (3), and therefore the high amount of Si in the produced alloys is from both the dross 2 (Figure 2) and also the aluminothermic reduction reaction.

2Al + Fe_2_O_3_ = 2Fe + Al_2_O_3_   ΔH (1500 °C) = −877.1 kJ/mol, (2)

2Al + 1.5SiO_2_ = 1.5Si + Al_2_O_3_   ΔH (1500 °C) = −262.5 kJ/mol. (3)

The small amounts of Ca and Mg in the produced metals are due to the distribution of these elements between the slag and metal. There is considerable CaO and, in lower levels, MgO in the reactive system, and these oxides can be partially reduced by Al due to their low chemical activities in the liquid metal, while their oxides have much higher chemical activities in the slags. Therefore, the mass transport of Ca and Mg occurs in the system via chemical reactions (4) and (5).

2Al + 3CaO = 3Ca + Al_2_O_3_   ΔH (1500 °C) = 250 kJ/mol, (4)

2Al + 3MgO = 3Mg + Al_2_O_3_   ΔH (1500 °C) = 142.5 kJ/mol. (5)

Comparing the compositions of Ca and Mg in the produced slags 3 and 4 indicates that more Ca has been transferred into the metal phase with increasing CaO in the charge. Meanwhile, the Mg in the metal phase has been decreased. This can be evaluated with regard to the slag thermochemistry and the effect of the CaO addition on the slag. When adding more CaO, as in exp. 4, this causes the increased chemical activity of the CaO in the slag and enhances the kinetics of reaction (4), yielding a metallic product with a higher Ca content. On the other hand, more CaO in the slag reduces the chemical activity of MgO due to the decreasing MgO concentration, which consequently decreases the MgO chemical activity. Hence, the extent of chemical reaction (5) is reduced and less Mg is transferred into the metal phase.

Figure 8 shows the microstructures of the produced metals in exp. 3 and 4. As can be seen, both metals have relatively similar phases with regard to the contrast; however, the amounts of the phases are different. The overall chemical composition of the phases measured for 4–5 very large areas (2 mm × 2 mm) and then averaged is given in Table 3. It is observed that the produced metals in the two experiments have very close chemical compositions of about 40–41% Mn, 31% Al, 21% Si, and 2.5–3.1% Fe. Excluding the C (from sample coating) and the minor oxygen concentration that may be due to sample surface oxidation, metal 4 has a two-times higher Ca concentration. The higher Ca concentration in the alloy 4 is attributed to the use of more CaO in the charge mixture in comparison with exp. 3, and this causes greater CaO chemical activity and the faster proceeding of the chemical reaction (4) in exp. 4.

The provided information in Table 3 shows that the microstructures of the produced Mn-Al-Si alloys in the two experiments 3 and 4 have similarities with regard to the type of coexisting phases, while their distribution is slightly different. In particular, phase 3, which is rich in Ca, is in a higher amount in metal 4 with a needle shape, while it is in a smaller size and irregular shape in metal 3. The dominant phase 1 in metal 4 has a larger size and most likely a higher amount than that in metal 3. However, the significantly higher amount of phase 2 in metal 3 than that in metal 4 provides relatively overall close chemical compositions for the two alloys, as the two phases 1 and 2 are the dominant phases.

The SEM/EDS analysis of the produced slags 3 and 4 indicated that they are calcium aluminate slags with minor impurities. It was found that slag 3 consists of two main phases of CaO·Al_2_O_3_ and CaO·2Al_2_O_3_, with an overall chemical composition of 24 wt.% CaO-76 wt.% Al_2_O_3_. The slag 4 was found to consist of CaO·Al_2_O_3_ and 12CaO·7Al_2_O_3_ phases. Obviously, the addition of more lime has affected the characteristics of the produced slags and the form of the calcium-aluminate phases, as expected with regard to the CaO·Al_2_O_3_ binary phase diagram.

As was previously mentioned, the produced metal has a low concentration of C, as shown in in Table 3 for the metal phase, which is due to sample coating. It is worth mentioning that in the Al-rich particles (separated from Al dross) and the utilized ferromanganese slag, we had insignificant amounts of C, and hence we did not expect this impurity in the produced metal and slag phases. We also expect the same for P, as again the two reactants are low in P, and in particular P in the original Al dross, where it was more distributed due to the fine milling. The concentration of O impurity is low, as shown in Figure 6b and Table 3, and we believe that O may be more concentrated on the metal sample surface due to surface oxidation. The sulfur content of the metal was insignificant and it was not detected by EDS analysis; hence, the S from ferromanganese slag may be more distributed in the produced slag phase, and probably some of it was lost due to evaporation.

## 4. Process Evaluation

The present experimental work indicates that it is possible to use a portion of the added metallic Al (pure or in dross) to conduct the aluminothermic reduction, and the rest of the Al is distributed in the metallic product. Hence, Al recovery depends on the MnO and SiO_2_ in the ferromanganese slag and also the amount of it. Meanwhile, it was indicated that there was a complete recovery of Mn and Si from the ferromanganese slag. Considering the results of this work and introducing a method for the aluminum dross and ferromanganese slag valorization, the process is shortly evaluated as follows.

### 4.1. Process Flexibility

**Figure 9:**
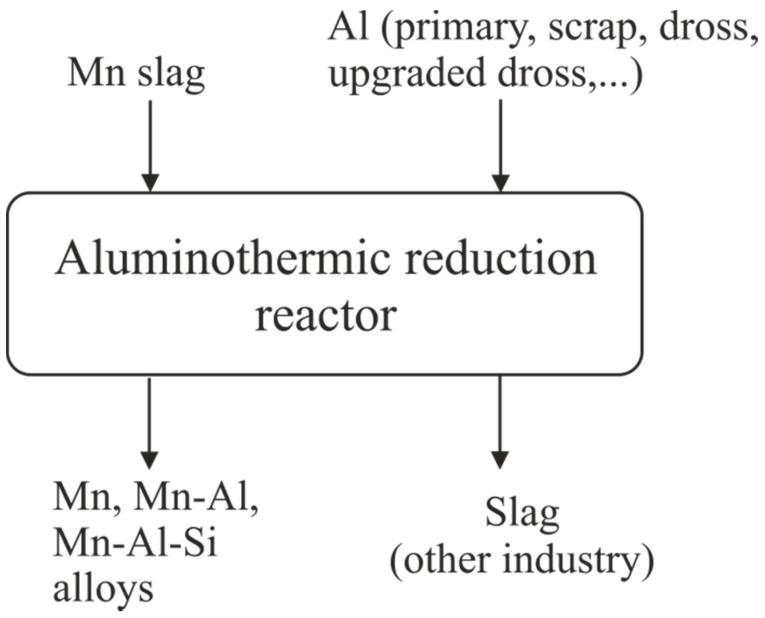
A simplified flowsheet of the process showing its flexibility.

The change of reactant materials from pure (metallic Al, MnO powder) to real industrial (Al dross, HCFeMn) materials indicates that it is possible to have a complete aluminothermic reduction process in both cases. Moreover, as a result of the reaction a complete manganese oxide reduction to metallic manganese was achieved in both cases, as illustrated in Figure 9. The present study shows that the process yields both Mn-Al and Mn-Al-Si alloys as the main product. The metallic product can be used in both the Al and steel industry—for instance, to produce Transformation Induced Plasticity (TRIP) and Twinning Induced Plasticity (TWIP) steels [32]. The produced calcium-aluminate slags that contain significant amounts of Al_2_O_3_ and CaO can be used easily in different industries, such as the cement, steelmaking, and aluminum industry, to recover alumina [33]. In the present study, pure Al and pure MnO-CaO slag were examined, yielding a high-purity Mn-Al alloy and a clean CaO-Al_2_O_3_ slag. Moreover, an upgraded Al dross, high in Al, was utilized to reduce industrial ferromanganese slag, and Mn-Al-Si alloys were obtained.

The applied process in this research is very flexible for recycling Al scrap, Al dross, etc., and producing valuable Mn and Mn-Al alloys. Obviously, the thermochemistry of the slag and metal system at elevated temperatures is very important, affecting the quality of the process products. In order to fully separate the reduced metal from the remaining slag, it is emphasized that the observed lab results are validated at a large scale. like the current ferromanganese process in which molten metal and slag are well separated due to their large density differences. A molten metal with 41%Al-32%Mn-22%Si-3%Fe and 2%Ca (as metals in trials 3 and 4 in Table 3) has an average density of 3.9 kg/cm^3^ (considering insignificant volume changes due to mixing), which is larger than the measured densities of calcium aluminate slags, that are in the range of 2.5–2.8 kg/cm^3^ at 1600 °C [34]. The lighter slag phase is obviously floating over the metal phase and can be separated easily in practice from the metal.

### 4.2. Energy Consumption

**Figure 10:**
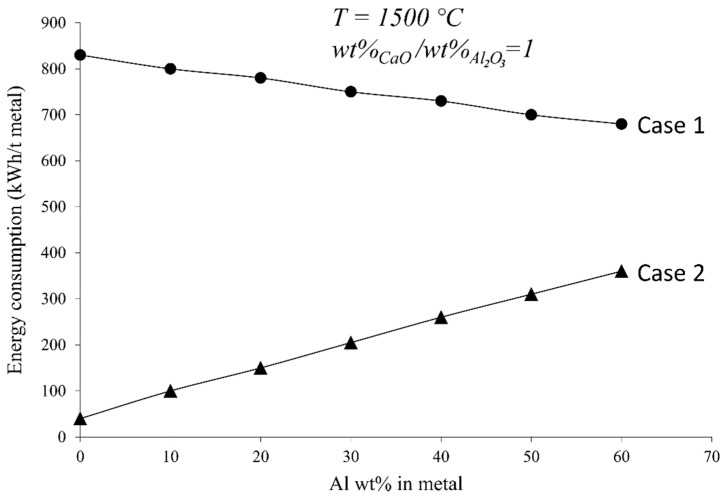
Calculated energy consumption for the Mn-Al alloy production from MnO-CaO slags. Case1: reactants are heated. Case 2: enthalpy of reactions is used to heat the reactants.

The process energy consumption for the presented aluminothermic reduction is mainly for heating the reactant materials to the high temperatures for the chemical reaction (1) to proceed. When the reaction is started, it is self-propagating and will continue until process completion. Hence, depending on the added reactants, the composition of the produced metal varies, as outlined above. The process energy consumption is, hence, very dependent on the characteristics of the reactants and their amounts. As the Al dross is a complex material that contains metallic Al and non-metallic components such as Al_2_O_3_, Al_4_C_3_, AlN, etc., it is difficult to provide highly representative numbers. Hence, the energy consumption was calculated for the case where Al interacted with MnO-CaO slag to produce Mn and Mn-Al alloys, according to the results in Section 3.2.1. Figure 10 indicates the calculated energy consumptions to produce unit mass of the metal for the case where the chemical composition of the produced slag is fixed (unity molar ratio of CaO/Al_2_O_3_ at 1773 K) and the amount of Al in the alloy varies. The calculations were completed using HSC Chemistry software version 7 for two cases—i.e., case 1, where all reactants are heated to the process temperature, and the heat generated by the reaction causes the temperature to rise in the reactor. For case 2, the enthalpy of the exothermic reaction (1) is completely utilized to heat up the charge as well. In case 2, it can be assumed that small amounts of the reactants are initially interact and then the rest of the charge is added so that the heat generated by the reactions is mostly utilized to heat up the more added cold charge to the reaction temperature. The latter case 2 is of course technology-dependent, and can be implemented in different ways in practice, which is beyond this study.

According to Figure 10, the aluminothermic process for Mn production presented in this work has a very low energy consumption, or, in another word, insignificant energy consumption. This low energy consumption is quite low if we compare it with the energy consumption for making alloys from pure metals, where 2000–3000 kWh/t [35] and 14,000–16,000 kWh/t [36] electric energy is used for Mn and Al production, respectively. Obviously, the energy consumption depends on the target alloy composition, and if the heat generated by the reaction (1) is not consumed to heat the materials, the energy consumption is slightly decreased with an increasing Al content. However, if the required heat for warming up the reactants is from the heat of reaction (1), the energy consumption is lower; however, it increases slightly with the increasing Al content of the target alloy. It is worth mentioning that the process energy consumption is lower in both cases, but the applied procedure in practice is more technology-dependent.

## 5. Conclusions

The aluminothermic reduction of MnO-containing slags was studied through using pure Al and two types of white Al dross, and the following results were obtained.

1. Pure Mn-Al alloys were obtained via the aluminothermic reduction of highly pure synthetic CaO-MnO slag by pure Al, and with Al dross from the primary Al production process.
2. The composition of the Mn-Al metal and the corresponding slag depends on the charge compositions and amounts.
3. Mn-Al-Si alloys were produced using upgraded industrial Al dross and industrial ferromanganese slag, and the metal composition is slightly dependent on additional flux (lime) addition. More Ca is transferred to the metal when the CaO content of slag is increased.
4. The composition and microstructure of the produced slag are very dependent on the charge mixture and can be easily engineered.
5. The outlined process is very flexible, and a variety of charge mixtures can be used.
6. The energy consumption of the process is low and is slightly affected by the target metal composition and the applied technology in practice regarding the energy savings.

## Figures and Tables

**Table 1:** The charge mixture details.

Exp. Number	Synthetic Slag (g)	HCFeMn Slag (g)	CaO Addition (g)	Al Metal (g)	Al Dross (g)
1	166	-	-	46	-
2	166	-	-	-	Dross 1 *: 69
3	-	49.5	11	-	Dross 2 **: 49.5
4	-	50.2	25.15	-	Dross 2 **: 50.2

*—Dross 1 with a particle size: 1–10 mm. **—Dross 2 with a particle size: 1.25–2 mm.

**Table 2:** Chemical composition of HCFeMn slag by XRF (wt.%).

Sample	MnO	SiO_2_	CaO	Al_2_O_3_	MgO	Fe_2_O_3_	K_2_O	BaO	SO_3_	Na_2_O	TiO_2_	SrO	Rest
FeMn slag	46.18	19.24	13.52	10.94	4.11	2.29	0.96	0.94	0.75	0.39	0.33	0.29	0.07

**Table 3:** Measured compositions for metals in exp. 3 and exp. 4 (wt.%).

	Al	Mn	Si	Mg	Fe	Ca	C	O
Metal 3 overall composition	41.33	31.12	21.80	0.59	2.54	0.62	1.60	0.41
Metal 4 overall composition	39.63	31.74	21.62	0.41	3.09	1.61	1.80	0.11
Phase 1	39.35	42.25	13.81	0.45	3.44	0.02	0.69	0.00
Phase 2	30.05	26.72	38.55	0.38	3.39	0.02	0.90	0.00
Phase 3	32.01	0.44	39.70	0.36	0.20	25.95	1.06	0.30
Phase 4	0.84	0.17	97.18	0.12	0.03	0.00	1.53	0.13

## Data Availability

Data available in a publicly accessible repository that does not issue DOIs. Publicly available datasets were analyzed in this study. This data can be found here: https://www.ntnu.edu/metpro.
